# Determining the relationship between breast cancer fear in women and levels of cyberchondria

**DOI:** 10.1590/1806-9282.20251804

**Published:** 2026-06-15

**Authors:** Neşe Kıskaç, Merve Özerdem, Refika Karahan, Dilan Orhan, Muharrem Kıskaç

**Affiliations:** 1 Istanbul Arel University, Faculty of Health Sciences, Department of Nursing – Istanbul, Turkey.; 2 Istanbul Gelisim University, Faculty of Health Science, Department of Nursing – Istanbul, Turkey.; 3 Acıbadem Fulya Hospital – Istanbul, Turkey.; 4 Biruni University, Faculty of Medicine, Department of Internal Medicine – Istanbul, Turkey.

**Keywords:** Breast cancer, Anxiety, Fear

## Abstract

**OBJECTIVE:**

The aim of this study was to examine the relationship between breast cancer fear and the level of cyberchondria in women.

**METHODS:**

This descriptive and cross-sectional study was conducted with 379 women aged 18 years and older. Data were collected using a Personal Information Form, the Cyberchondria Severity Scale-Short Form, and the Breast Cancer Fear Scale. Statistical analyses were performed using IBM Statistical Package for the Social Sciences version 26.0.

**RESULTS:**

The participants’ mean total score on the Cyberchondria Severity Scale-Short Form was 33.73±10.72 (moderate level), while the mean total score on the Breast Cancer Fear Scale was 24.37±9.11 (high level). Significant associations were found between the Cyberchondria Severity Scale-Short Form total score and participants’ educational status, menopausal status, and practice of breast self-examination, as well as between the Breast Cancer Fear Scale total score and history of clinical breast examination (p<0.05). A positive significant correlation was observed between the Cyberchondria Severity Scale-Short Form total score and the Breast Cancer Fear Scale total score (r=0.464; p<0.05).

**CONCLUSION:**

Nurses should promote health literacy and guide individuals toward reliable sources, employing an interdisciplinary approach to manage patient fears. By increasing awareness of regular screenings and breast self-examinations, nurses play a key role in mitigating anxiety and fear among high-risk women.

## INTRODUCTION

Cancer remains a global public health priority as the second leading cause of death, with incidences rising due to aging populations and lifestyle factors^1,2^. Among women, breast cancer is the most prevalent malignancy, accounting for 24% of all female cancer cases with 2.3 million annual diagnoses^3^. Beyond physiological effects, a breast cancer diagnosis significantly impairs psychological well-being, often triggering fear, anxiety, and uncertainty regarding recurrence. These fatalistic perceptions influence coping mechanisms and health behaviors, frequently driving individuals to seek information^4^. In the digital era, the internet is a primary source of health information; however, excessive searching can lead to cyberchondria, an escalation of anxiety resulting from online health inquiries^5^. Exposure to inaccurate digital information can undermine treatment trust and provoke unnecessary panic^6–11^. Consequently, nurses carry a substantial responsibility to provide professional support, promote early detection, and guide patients toward reliable sources to mitigate anxiety^4^. This study aims to examine the relationship between breast cancer fear and cyberchondria levels in women. Understanding this interaction is crucial for improving health behaviors and guiding healthcare professionals in developing strategies to manage the psychological impact of the disease, ultimately enhancing the quality of life for those at risk or diagnosed.

## METHODS

### Study design

This research was designed as a descriptive and cross-sectional study.

### Sample

The study's sample size was calculated as 377 based on a 95%CI and a 5% margin of error, with 379 women ultimately participating. Utilizing snowball sampling, participants were recruited via telephone directories. Data collection was conducted digitally through Google Forms sent to the participants’ mobile devices.

### Data collection procedure

Data were collected online via a self-administered questionnaire between January 1 and April 9, 2025.

### Instruments

Study data were obtained using a Personal Information Form, the Cyberchondria Severity Scale-Short Form (CSS-SF), and the Breast Cancer Fear Scale (BCFS). Guidelines on breast cancer list the individual's age, age at first menstruation, menopausal status, oral contraceptive use, body mass index (BMI), age at first birth, family history of breast cancer, hormone replacement therapy, and exercise as risk factors for breast cancer. Therefore, the personal data collection form for women collects information specified as risk factors in the guidelines^12,13^.

### Cyberchondria Severity Scale-Short Form

Cyberchondria involves heightened anxiety from excessive online health searching. We used the 12-item Short Form (CSS-SF), developed by McElroy and Shevlin and revised by McElroy et al.^14,15^. Validated in Turkish^16,17^, it assesses four subscales on a five-point Likert scale (score range: 12–60). Higher scores indicate greater severity. This study found a Cronbach's alpha of 0.89.

### Breast Cancer Fear Scale

The 8-item BCFS, developed by Champion et al.^18^ and validated in Turkish by Seçginli^19^, assesses emotional responses to breast cancer. Scores (8–40) categorize fear as low, moderate, or high. In this study, Cronbach's alpha was 0.93, indicating excellent internal consistency.

### Data analysis

Data were analyzed using IBM Statistical Package for the Social Sciences 26.0. To compare groups, t-test/analysis of variance (for normal distribution) and Mann-Whitney U/Kruskal-Wallis (for non-normal distribution) were utilized. Relationships were assessed via Pearson/Spearman correlations, while multiple regression controlled for demographic variables. Statistical significance was set at p<0.05 with 95%CI.

### Ethical considerations

Ethical approval was granted by Istanbul Gelisim University Ethics Committee (No: 2024-20-10; Date: 19 Dec 2024). All participants provided written informed consent after being briefed on the study's purpose. This research adhered to the Declaration of Helsinki and national ethical standards.

## RESULTS

The relationships between participants’ sociodemographic characteristics, the total scores of the CSS-SF, and the BCFS are presented in Tables 1 and 2. The mean total CSS-SF score of the participants was 33.73±10.72, indicating a moderate level of cyberchondria. The mean total BCFS score was 24.37±9.11, reflecting a high level of breast cancer fear. Significant associations were found between the CSS-SF total score and participants’ educational status, menopausal status, and practice of breast self-examination, as well as between the BCFS total score and history of undergoing clinical breast examination (p<0.05) (Tables 1 and 2). CSS-SF scores significantly associated with educational status, menopausal status, and breast self-examination (p<0.05). To control for these confounders, multiple regression was performed. Results showed that independent of education (p=0.665), menopause (p=0.735), and self-examination (p=0.077), a moderate positive correlation persisted between total BCFS and CSS-SF scores (r=0.464; p<0.05).

**Table 1:** Relationship between participants’ sociodemographic characteristics and the total scores of the Cyberchondria Severity Scale-Short Form and the Breast Cancer Fear Scale (n=379).

		n	%	Total CSS-SF score	p	Total BCFS score	p
Age (years)		30.65±11.35					
	≥30	150	39.6	33.35±11.29	0.577	24.09±9.44	0.652
	<30	228	60.4	33.98±10.37		24.53±8.90	
Marital status							
	Single	228	60.2	34.18±10.06	0.315	24.39±9.06	0.976
	Married	151	39.8	33.05±11.65		24.36±9.21	
Educational level							
	Primary and secondary school	45	11.9	30.13±11.08	**0.006**	21.98±10.22	0.254
	High school	84	22.2	32.00±12.02		24.08±9.71	
	Undergraduate	222	58.6	34.65±10.25		24.94±8.75	
	Postgraduate	28	7.4	37.43±7.25		24.61±7.86	
Age at menarche		13.36±1.87					
	≥13	266	70.2	33.50±10.71	0.510	24.19±9.16	0.549
	<13	113	29.8	34.29±10.77		24.81±9.00	
Menopausal status							
	No	346	91.3	34.12±10.48	**0.023**	24.52±9.08	0.324
	Yes	33	8.7	29.70±12.43		22.88±9.35	
Age at menopause		46.79±4.65 (n=33)					
	≥46	20	60.6	29.62±12.32	0.904	23.38±9.66	0.678
	<46	13	39.4	30.15±12.66		22.00±8.75	
Age at first birth		24.51±4.47 (n=139)					
	≥24	78	56.1	33.41±12.23	0.916	24.56±9.78	0.710
	<24	61	43.9	33.62±11.15		23.95±9.43	
Use of oral contraceptives							
	No	338	89.2	33.78±10.73	0.805	24.61±9.05	0.150
	Yes	41	10.8	33.34±10.77		22.44±9.43	
Body mass ındex		24.89±4.49					
	≥24	194	51.2	32.99±11.37	0.167	24.07±9.70	0.509
	<24	185	48.8	34.51±9.97		24.69±8.46	

Descriptive statistical methods (mean, standard deviation, frequency, and percentage) were used. For group comparisons, Student's t-test and Mann-Whitney U test were applied as appropriate. CSS-SF: Cyberchondria Severity Scale-Short Form; BCFS: Breast Cancer Fear Scale. Bold text indicates a statistically significant difference between the variables (p<0.05).

**Table 2:** Relationship between participants’ sociodemographic characteristics and the total scores of the Cyberchondria Severity Scale-Short Form and the Breast Cancer Fear Scale (n=379) continue.

		n	%	Total CSS-SF score	P	Total BCFS score	p
Hormone replacement therapy (HRT) use							
	No	360	95.0	33.80±10.66	0.615	24.32±9.12	0.626
	Yes	19	5.0	32.53±12.10		25.37±9.02	
Regular exercise status							
	No	265	69.9	34.02±10.84	0.430	24.44±9.17	0.838
	Yes	114	30.1	33.07±10.46		24.23±9.00	
History of breast-related health problems							
	No	348	91.8	33.76±10.71	0.865	24.45±9.11	0.613
	Yes	31	8.2	33.42±10.98		23.58±9.17	
Family history of breast cancer							
	No	306	80.7	33.53±10.74	0.456	23.92±9.01	0.470
	Yes	73	19.3	34.58±10.69		26.27±9.31	
Practice of breast self-examination (BSE)							
	No	144	38.0	31.86±10.28	**0.008**	24.33±8.74	0.936
	Yes	235	62.0	34.88±10.85		24.40±9.34	
History of undergoing mammography							
	No	306	80.7	33.74±10.72	0.985	24.37±9.21	0.981
	Yes	73	19.3	33.71±10.79		24.40±8.71	
History of receiving education about breast cancer							
	No	263	69.4	33.46±10.63	0.456	24.56±9.01	0.562
	Yes	116	30.6	34.35±10.94		23.97±9.35	
Total CSS-SF score		33.73±10.72 (min:12–max:60)					
Excessiveness		10.24±3.59 (min:3–max:15)					
Distress score		8.28±3.37 (min:3–max:15)					
Compulsions		8.95±3.18 (min:3–max:15)					
Difficulty score		6.26±3.07 (min:3–max:15)					
Total BCFS score		24.37±9.11 (min:8–max:40)					

Descriptive statistical methods (mean, standard deviation, frequency, and percentage) were used. For group comparisons, Student's t-test and Mann-Whitney U test were applied as appropriate. CSS-SF: Cyberchondria Severity Scale-Short Form; BCFS: Breast Cancer Fear Scale; HRT: Hormone replacement therapy; BSE: breast self-examination. Bold text indicates a statistically significant difference between the variables (p<0.05).

A statistically significant positive correlation was found between the CSS-SF total score and the BCFS total score (r=0.464; p<0.05). Furthermore, a significant positive correlation was also found between the BCFS and CSS-SF subdimensions (Table 3).

**Table 3:** Relationship between participants’ age, body mass index, age at first birth, age at menopause, age at menarche, total Cyberchondria Severity Scale-Short Form score and subdimensions, and total Breast Cancer Fear Scale score (n=379).

		Excessiveness	Compulsions	Distress	Reassurance	CSS-SF	BCFS
Age	r	-0.120	0.030	0.017	0.012	-0.022	-0.024
	p	**0.020**	0.564	0.743	0.816	0.665	0.645
Age at menarche	r	-0.070	-0.092	-0.057	0.014	-0.066	0.013
	p	0.171	0.074	0.270	0.792	0.203	0.804
Age at menopause	r	-0.080	0.099	-0.018	-0.053	-0.019	-0.056
	p	0.653	0.576	0.920	0.765	0.916	0.753
Age at first birth	r	-0.035	-0.038	-0.049	-0.136	-0.076	-0.054
	p	0.680	0.661	0.565	0.110	0.374	0.527
BMI	r	-0.180	0.014	-0.071	0.009	-0.074	-0.004
	p	**0.000**	0.780	0.170	0.856	0.149	0.935
Excessiveness	r	1	0.533	0.650	0.365	0.801	0.286
	p		**0.000**	**0.000**	**0.000**	**0.000**	**0.000**
Compulsions	r	0.533	1	0.630	0.560	0.841	0.436
	p	**0.000**		**0.000**	**0.000**	**0.000**	**0.000**
Distress	r	0.650	0.630	1	0.516	0.861	0.382
	p	**0.000**	**0.000**		**0.000**	**0.000**	**0.000**
Reassurance	r	0.365	0.560	0.516	1	0.738	0.409
	p	**0.000**	**0.000**	**0.000**		**0.000**	**0.000**
CSS-SF	r	0.801	0.841	0.861	0.738	1	0.464
	p	**0.000**	**0.000**	**0.000**	**0.000**		**0.000**
BCFS	r	0.286	0.436	0.382	0.409	0.464	1
	p	**0.000**	**0.000**	**0.000**	**0.000**	**0.000**	

Pearson's and Spearman's correlation analyses. CSS-SF: Cyberchondria Severity Scale-Short Form; BCFS: Breast Cancer Fear Scale; BMI: body mass index. Bold text indicates a statistically significant difference between the variables (p<0.05).

## DISCUSSION

This study aims to determine the relationship between breast cancer fear and cyberchondria in women, and to identify the associated individual characteristics. A review of the literature reveals a limited number of studies examining the link between cancer and cyberchondria; however, no research has been found specifically investigating the relationship between breast cancer fear and cyberchondria among women. Therefore, the findings of this study will be discussed in the context of the limited existing literature.

This study identified a moderate positive correlation between breast cancer fear and cyberchondria (r=0.464; p<0.05), with the "compulsions" subscale showing the strongest association (r=0.436). Findings suggest online research exacerbates fear, aligning with literature. Coşkun et al. noted that while extensive health-related searching increases awareness, it induces confusion and correlates with compulsive information-seeking^20^. Similarly, Topkara Sucu et al. found significantly higher cyberchondria severity in adolescents with Polycystic Ovary Syndrome compared to healthy controls^21^. Öncü et al. also reported a significant correlation between Human Papillomavirus 16/18 positivity and cyberchondria levels^22^. Consistent with these studies, our results indicate that medical conditions increase cyberchondria severity; although this may enhance awareness, it simultaneously triggers confusion, ultimately escalating anxiety and fear.

In both the current study and the study by Öncü et al.^22^, cyberchondria severity was found to be significantly higher among individuals with higher education levels. It is considered that those with higher education may possess greater awareness of risk factors, which potentially accounts for their elevated cyberchondria levels. Conversely, in the study by Coşkun et al., although no significant correlation was found between education level and cyberchondria severity, scores were notably higher among individuals with lower education levels^20^. This discrepancy may be attributed to the fact that lower levels of health literacy or awareness might lead individuals to engage in more extensive online research.

The significantly lower CSS-SF scores in postmenopausal women may be attributed to unique psychosocial stressors of this stage. Citing Zangirolami-Raimundo et al., who highlighted depression during the climacteric period, it is suggested that psychological shifts across different life stages influence health information-seeking behaviors^23^.

Participants practicing breast self-examination exhibited significantly higher CSS-SF scores. Research indicates that emotional factors and fears can modify individuals’ clinical outlooks and health-related behaviors. In this study, it is suggested that the fear experienced by women acted as a driver, altering their health behaviors and increasing the frequency of Breast Self-Examination practice^24,25^.

Although not always reaching statistical significance across all parameters, cyberchondria scores were found to be higher among participants under the age of 30, those who were premenopausal (p<0.05), and those who performed breast self-examination (p<0.05). Additionally, a low-level significant correlation was identified between age and the "excessiveness" subdimension of the cyberchondria scale. These findings suggest that individuals under the age of 30 engage in more intensive health-related online research. Consequently, increased awareness regarding breast cancer may have prompted a higher frequency of breast self-examination behaviors. Multiple regression analysis conducted in this study revealed that, independent of demographic variables, there is a moderate, positive, and significant relationship between cyberchondria scores and breast cancer fear. In alignment with the existing literature, this study demonstrates that the severity of cyberchondria exacerbates the fear of breast cancer.

### Limitations

This study has several limitations. The use of snowball sampling and an online survey may limit the generalizability of the findings to the broader population of women, potentially over-representing those with higher digital literacy or health anxiety. The cross-sectional design cannot establish causality between breast cancer fear and cyberchondria. Future longitudinal studies with more representative sampling methods are needed to confirm these associations and explore causal pathways.

## CONCLUSION

This study demonstrates a significant relationship between breast cancer fear and cyberchondria levels among women. Particularly in individuals with high education and awareness levels, existing medical conditions or high-risk factors prompt more intensive research; this, in turn, may lead to confusion and further escalate anxiety and stress. These findings underscore the necessity of strengthening digital health literacy within nursing practices. Nurses should educate individuals on health literacy and direct them toward reliable information sources. Furthermore, an interdisciplinary approach involving other healthcare professionals is essential to help individuals manage their fears effectively. By raising awareness about regular screenings and breast self-examination among high-risk women, nurses can play a pivotal role in supporting patients in managing their anxiety and fear.

## Data Availability

The datasets generated and/or analyzed during the current study are available from the corresponding author upon reasonable request.
